# Posterior Meniscal Root Repair with Epidural Needle through the Combination of Arthroscopic Portals and Transtibial Tunnel

**DOI:** 10.1055/s-0044-1790214

**Published:** 2024-12-21

**Authors:** Eiji Rafael Nakahashi, Igor Leal Clemente Lemes, Mauro Batista Albano, Edmar Stieven Filho, Mario Massatomo Namba, Carolline Popovicz Nunes

**Affiliations:** 1 Grupo de Cirurgia do Joelho e Trauma do Esporte, Complexo Hospital do Trabalhador, Universidade Federal do Paraná, Curitiba, PR, Brasil

**Keywords:** meniscus, surgical procedures, suture techniques, tibial meniscus injuries

## Abstract

Posterior meniscal root repair is an expensive procedure because its performance often requires the use of specific devices. This issue is a limiting factor, especially in the public health system. Given this context, the development of alternative methods to treat these injuries became necessary. Among the available options, the technique combining the use of anterior portals and a tibial bone tunnel with an epidural needle has been proven to be effective and relevant due to its low cost. The present study aimed to provide technical guidance and suggestions to increase the success rate of this procedure, to enable its performance by knee surgeons in low-resource settings.

## Introduction

Among meniscal injuries, those to the root are significant because of the development of gonarthrosis, as they compromise the biomechanical function of the menisci to a greater extent than simple meniscal injuries. 1 Biomechanical studies 1 have demonstrated that meniscal root injuries behave like total meniscectomy. As such, there is a loss of the protective function of the menisci, resulting in a series of degenerative changes due to joint overload. 2 3 In contrast, some authors 4 have shown that repairing these injuries, especially in younger patients with few degenerative abnormalities, can change their evolution and prognosis.

In this context, throughout the years, the development of equipment and instruments to treat root lesions sought to achieve a less invasive and faster technique. 5 6 However, the high cost of the materials available makes its widespread use unfeasible in public hospitals. Therefore, we tried to develop a cost-effective technique combining an epidural needle, anterior arthroscopic portals, and a transtibial bone tunnel. This technique proved to be very useful in the context of the lack of proper materials to perform this procedure in the hospitals of the Brazilian Unified Health System (Sistema Único de Saúde, SUS, in Portuguese). The present study aimed to provide technical guidance and suggestions to increase the procedural success rate, enabling its performance by knee surgeons in locations with limited resources.

## Description of the Surgical Technique

Place the patient in the supine position after spinal anesthesia. Put a side post with a protective cushion to assist during arthroscopic inspection maneuvers. The use of a tourniquet and leg positioning are at the surgeon's discretion. The leg may remain suspended or placed on the operating table during surgery, taking care to enable knee flexion and adjusting the height of the operating table to avoid contamination. Perform standard routine orthopedic preparations and sterile drape placement.

### Steps of the Surgical Technique

The anterolateral (AL) portal is slightly superior (high and tight). Create the anteromedial (AM) portal under direct visualization with the aid of a 16-G catheter (Abbocath, Abbott Laboratories, Chicago, IL, United States) to verify the ideal height to reach the posterior region of the knee. Then, inspect the joint to evaluate all compartments before beginning meniscal repair. Below, we will detail the technique in steps.

First step: After confirming the root injury and its potential repair, begin preparation with intercondylar region debridement. Use a soft-tissue shaver and bone curette to facilitate access to the region of the posterior root of the medial or lateral meniscus. If necessary, perform the pie-crusting technique in the medial collateral ligament (MCL) to gain additional space. The Gillquist maneuver (transnotch view), introducing the arthroscope through the AL portal between the posterior cruciate ligament (PCL) and the lateral aspect of the medial femoral condyle (MFC), enables optimal visualization of the posterior portion. An accessory transpatellar portal may be opened in half the thickness of the patellar tendon after checking the ideal height to enable instrumentation in the posterior region of the knee.

**Fig. 1:**
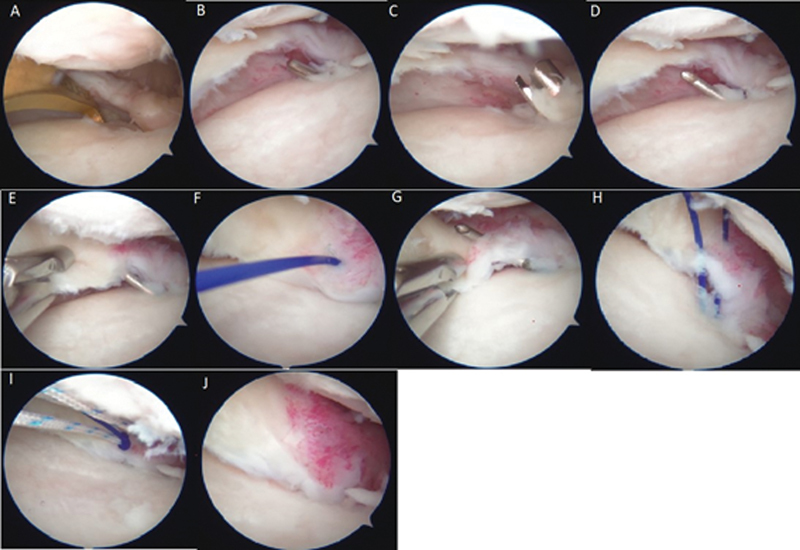
Arthroscopic visualization of the reattachment stages. ( **A** ) Assessment of the possibility of suturing and positioning the tibial guide; ( **B** ) guidewire positioning; ( **C** ) creation of a bone tunnel with a drill; ( **D** ) introduction and adjustment of the epidural needle inside the tunnel; ( **E** ) meniscus perforation with a needle; ( **F** ) introduction of the polypropylene (transport) suture; ( **G** ) meniscus perforation in a new position; ( **H** ) visualization of transport and positioning wires; ( **I** ) tying of polypropylene threads into the definitive suture; and ( **J** ) traction of the definitive polyester suture through the bone tunnel.

**Fig. 2:**
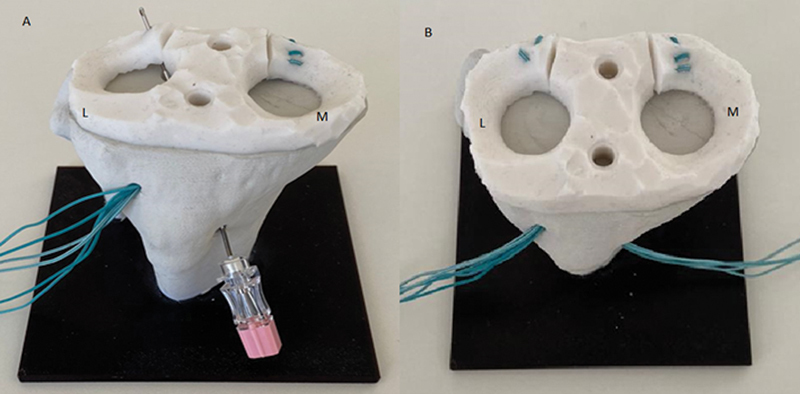
Final aspect of the lateral root repair in a synthetic model. Note that the entry point is at the anteromedial cortex.

**Fig. 3:**
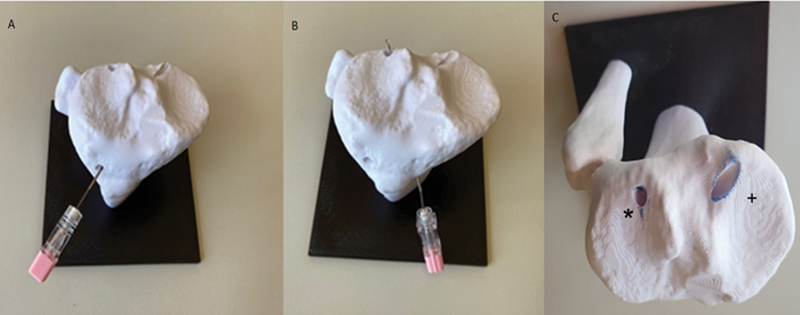
Shape and position of the tunnels concerning the attack angle in the vertical plane to the tibial surface. ( **A** ) Oval-shaped orifice (lower inclination); ( **B** ) circular-shaped orifice (higher inclination); and ( **C** ) oval-shaped orifice (+); circular orifice (*).

Second step: Position the ACL guide as planned ( Fig. 1A ). The intra-articular support end (target) must have a low profile. Other types of guide increase the risk of chondral injury and make positioning difficult. We recommend making the entry point of the tibial tunnel on the opposite side of the injury ( Figs. 2 , 3 ). Therefore, for injuries at the posterior root of the medial meniscus, we suggest an entry point in the AL cortex of the proximal tibia. In lateral meniscal root injuries, we recommend an entry point in the AM cortex. This arrangement enables a higher attack angle to position the needle in the posterior horn of the meniscus. Thus, it is easier to reach the ideal location regarding repair positioning, since many lesions present edge fragmentation or irregularity.

Third step: The inclination angle of the guide influences the work area created to reach the desired location with the epidural needle ( Fig. 3A,B ). The smaller the inclination (attack) angle, the larger the area created. An angle reduction results in an oval-shaped exit hole, enabling sutures of up to 1 cm when using 4.5-mm drills. In contrast, an increased angle makes the hole shape closer to that of a circle. For the same reason, an increased angle may damage the cartilage and adjacent structures. Next, position a 2.5-mm guidewire and check its location ( Fig. 1B ). If the site is good, proceed to drill with a cannulated device ( Fig. 1C ). Drill the articular portion of the tibia at low rotation or with manually-controlled rotation.

Fourth step: Choose the drill diameter to create the transtibial tunnel as explained in the third step. Increasing the diameter will result in a larger work area, but it will present a greater potential for chondral and adjacent structure damages. We suggest drilling with a 6- or 7-mm cannulated drill trying to reproduce the anatomy area of the medial and lateral posterior meniscal roots, respectively. 7 The surgeon must be aware not only of the inclination angle of the tibial guide, but also of the chosen drill diameter.

**Fig. 4:**
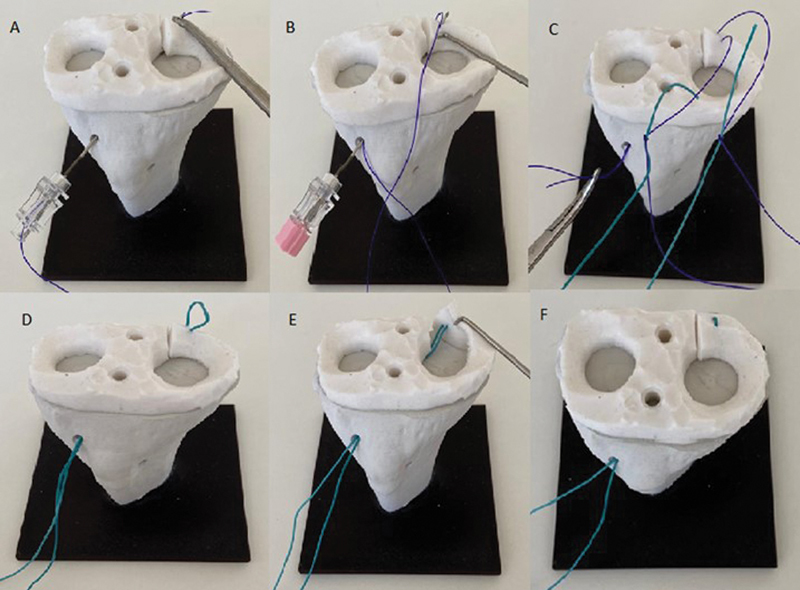
Demonstration of the technique for the medial meniscus of the right knee. ( **A,B** ) Needle insertion and introduction of two transport (polypropylene) sutures; ( **C** ) fixation of the definitive (polyester) suture at the ends of the transport threads; ( **D** ) traction of the transport threads through the distal portions in the tibial orifice; and ( **E,F** ) final aspect of the repair of the posterior horn root of the medial meniscus.

Fifth step: Introduce the epidural needle (18 Gx150 mm) into the tunnel ( Figs. 4A,B , 1D,G ). This needle is malleable and suitable for passing through oblique long tunnels. A probe or grasping forceps may assist meniscal perforation by adjusting the position of the meniscal edge per the traction generated towards the tunnel or its angulation. This maneuver prevents the edge from rising during needle passage. Meniscal manipulation with these instruments enables the change in the needle penetration angle, adjusting the width of the suture. Controlling the orientation of the needle bevel can increase the suture range by rotating it 180° to the first needle pass, always keeping it supported on the tunnel walls. Furthermore, perform drilling with the mandrel inside it to avoid the entry of meniscal tissue and, as a result, inadequate drilling.

Sixth step: After perforation, we suggest passing the #2 polypropylene suture inside the needle ( Fig. 4A ). Next, capture the suture thread at one of the previous portals and bring it out of the portal ( Fig. 4B ). Attach a repair to the distal end of the wire. Repeat this step to make another suture on the same meniscal edge ( Fig. 4C ). Tie the two proximal ends of the polypropylene suture brought to the portals with a #2 polyester thread ( Figs. 1C , 4C ). Apply light traction to the distal ends of the transport wires at the entrance of the tibial tunnel. At the end, a suture with a “U” configuration ( Figs. 4D–F , 2B ) requires fixation on the tibial cortex. We suggest making at least one more suture to increase resistance and divide local stress, reducing the chance of failure.

Seventh step: Perform distal fixation depending on material availability. The options include a washer, a pole, high-resistance thread anchor, and a tibial transosseous suture. In this last step, the surgeon must visualize the accommodation position and the re-establishment of the meniscal anatomy before tightening the knot ( Fig. 1J ) with the knee at a 90° flexion.

At the end, perform a new inspection and remove any debris. Then, test meniscal stability during flexion-extension for the range of motion from 0 to 90° of flexion. Finally, close the approach at your discretion.

**Fig. 5:**
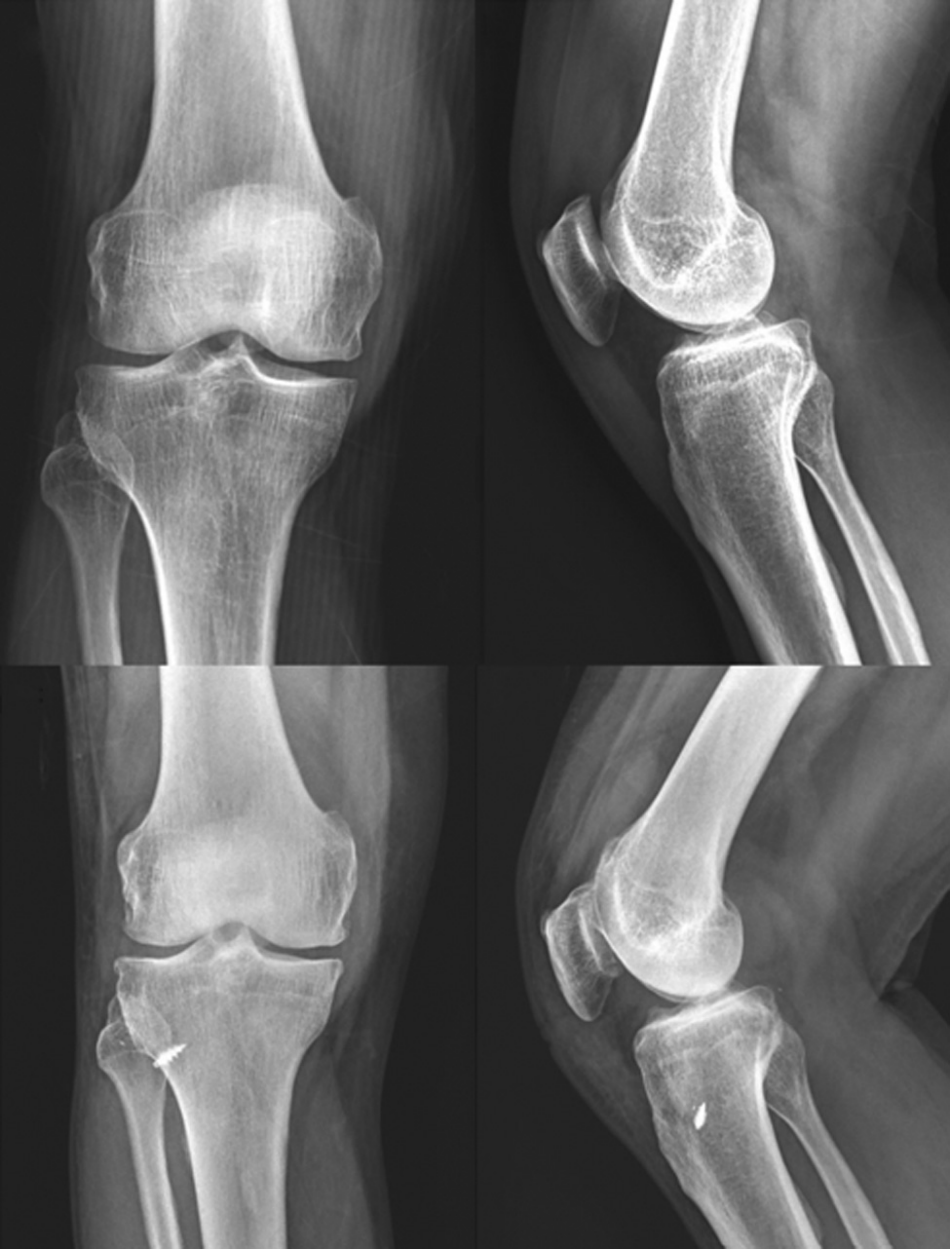
Preoperative and postoperative follow-up radiographs.

Fig. 5 shows the initial and last radiographs of a case of repair of the posterior root of the medial meniscus from a right knee. In this case, we fixated the high-strength suture distally using a bone anchor.

## Final Considerations

The posterior meniscal root repair technique with an epidural needle and combined anterior portals and a transtibial tunnel proved to be feasible and accessible. Its use can eliminate the need for posterior portals, and the strategies herein described enable the adequate restitution of meniscal anatomy. In our practice, we have performed twelve posterior meniscal root repairs using this technique. Of these, nine were on the medial meniscus, and three, on the lateral meniscus. No case combined the repair with any concomitant procedure (ligament reconstruction or osteotomy). Furthermore, no re-approach was necessary during the 12-month follow-up, and the patients showed satisfactory progress. However, we did not use any assessment score during the follow-up period, and further studies are required to assess the long-term outcomes.

It is worth highlighting that the technique requires surgical experience with arthroscopic maneuvers in the posterior region of the knee, especially in cases of constriction of the compromised compartment. The difficulty in manipulating instruments in this space can increase surgical time. The potential ways to make the technique easier include using a pneumatic tourniquet to reduce intra-articular bleeding and facilitate joint visualization. In subjects with compartment constriction, pie-crusting of the MCL can increase the working space. Furthermore, we recommend using a guide with a flat target to facilitate positioning and reduce the chance of iatrogenic injuries to the adjacent cartilage. Other potential complications include meniscal laceration, which can occur after pulling the suture thread through the tibial orifice and lead to repair failure, and the persistence of postrepair meniscal extrusion. To correct this last complication, make a centralization suture to re-establish the congruity of the meniscal tissue.

The present study aimed to provide suggestions and technical details to increase the success rate of posterior meniscal root repair using low-cost materials. Thus, we have an effective option with accessible materials to use when resources are limited and in unforeseen situations, avoiding the need for an additional procedure due to a lack of specific surgical instruments.
