# Supplementary material for: Posterior Meniscal Root Repair with Epidural Needle through the Combination of Arthroscopic Portals and Transtibial Tunnel
Source: Rev Bras Ortop (Sao Paulo). 2024 Dec 21;59(6):e997–e1002. doi: 10.1055/s-0044-1790214 (PMC11663058; doi:10.1055/s-0044-1790214)
Supplement: Related texts

# Research article: Reparo das raízes meniscais posteriores com agulha peridural por meio da combinação de portais artroscópicos e túnel transtibial

**Keywords:** lesões do menisco tibial, menisco, procedimento cirúrgico, técnicas de sutura

## Abstract

O reparo de raízes meniscais posteriores é um procedimento considerado de alto custo pelo uso habitual de dispositivos específicos para sua realização. Essa situação acaba se tornando um fator limitante, principalmente no cenário do sistema público de saúde. Diante desse contexto, tornou-se necessário o desenvolvimento de métodos alternativos para o tratamento dessas lesões. Entre as opções possíveis, a técnica que combina o uso de portais anteriores e túnel ósseo tibial com a utilização de agulha peridural mostrou-se eficaz e de grande relevância devido ao seu baixo custo. Este estudo tem como finalidade trazer orientações técnicas e sugestões para aumentar a taxa de sucesso desse procedimento e possibilitar sua realização por cirurgiões de joelho em locais com recursos limitados.

## Introdução

Entre as lesões meniscais, as da raiz são de grande importância no que diz respeito ao desenvolvimento de gonartrose, pois comprometem a função biomecânica dos meniscos em maior extensão do que lesões meniscais simples. 1 Estudos biomecânicos 1 demonstraram que lesões da raiz meniscal se comportam como casos de meniscectomia total. Deste modo, há perda da função protetora dos meniscos, e dá-se início a uma série de alterações degenerativas por sobrecarga articular. 2 3 Por outro lado, alguns autores 4 já demonstraram que o reparo, indicado principalmente em pacientes mais jovens e com poucas alterações degenerativas, pode alterar a evolução e prognóstico dessas lesões.

Nesse contexto, com o passar dos anos, foram desenvolvidos equipamentos e instrumentais para o tratamento das lesões de raiz, na tentativa de alcançar uma técnica menos invasiva e de realização mais rápida. 5 6 Contudo, o alto custo associado aos materiais disponíveis inviabiliza a difusão do seu uso em hospitais públicos. Dessa forma, buscou-se desenvolver uma técnica com boa relação custo-benefício por meio do uso combinado de agulha epidural, portais artroscópicos anteriores e um túnel ósseo transtibial. Essa técnica demonstrou ser de grande utilidade na ausência de material próprio para a realização deste procedimento em hospitais do Sistema Único de Saúde (SUS). Este estudo tem como finalidade trazer orientações técnicas e sugestões para aumentar a taxa de sucesso desse procedimento e possibilitar sua realização por cirurgiões de joelho em locais com recursos limitados.

## Descrição da Técnica Cirúrgica

O paciente é posicionado em decúbito dorsal após raquianestesia. Posiciona-se um poste lateral com coxim de proteção para auxílio durante as manobras de inspeção artroscópica. O uso do garrote e o posicionamento da perna ficam a critério do cirurgião. A perna pode ser deixada suspensa ou sobre a mesa operatória durante a cirurgia, com o cuidado de permitir flexão do joelho e ajuste da altura da mesa cirúrgica para evitar contaminação. Os preparos ortopédicos de rotina e o posicionamento de campos estéreis são realizados de modo convencional.

## Etapas da Técnica Operatória

O portal anterolateral (AL) é aberto em posição ligeiramente superior ( *high and tight* ). A confecção do portal anteromedial (AM) é feita sob visualização direta, com o auxílio de um catéter de calibre 16 (Abbocath, Abbott Laboratories, Chicago, IL, Estados Unidos) para a verificação da altura ideal, visando atingir a região posterior do joelho. A inspeção articular é então realizada para a avaliação de todos os compartimentos antes do início do reparo meniscal. Em seguida, a técnica será detalhada em etapas.

Primeiro passo: após a confirmação da lesão da raiz e da possibilidade de reparo, inicia-se o preparo com o debridamento da região intercondilar. Para isso, pode-se utilizar o microdebridador de partes moles e a cureta óssea para facilitar o acesso à região da raiz posterior do menisco medial ou lateral. Em caso de necessidade, pode-se realizar a técnica de *pie-crusting* do ligamento colateral medial (LCM) para ganho de espaço adicional. A visualização ideal da porção posterior pode ser obtida por meio da manobra de Gillquist ( *transnotch view* ), em que o artroscópio é introduzido pelo portal AL entre o ligamento cruzado posterior (LCP) e a face lateral do côndilo femoral medial (CFM). Um portal transpatelar acessório pode ser criado na metade da espessura do tendão patelar, após prévia checagem da altura ideal, para permitir instrumentação na região posterior do joelho.

Segundo passo: em seguida, coloca-se o guia de LCA na posição planejada ( Fig. 1A ), devendo este ter a extremidade de apoio intra-articular (alvo) com perfil baixo. Outros tipos de guia aumentam o risco de lesão condral e dificultam o posicionamento. Recomenda-se que o ponto de entrada do túnel tibial seja realizado no lado oposto ao da lesão ( Figs. 2 , 3 ). Assim, para lesões da raiz posterior do menisco medial, sugere-se que a entrada seja feita na cortical AL da tíbia proximal. Já em lesões de raiz meniscal lateral preconiza-se que o ponto de entrada seja na cortical AM. Essa disposição permite um maior ângulo de ataque para o posicionamento da agulha no corno posterior do menisco. Com isso, para é mais fácil atingir o local julgado como ideal quanto ao posicionamento do reparo, pois muitas lesões apresentam-se com fragmentação ou irregularidade em seus bordos.

**Fig. 1:** Visualização artroscópica das etapas da reinserção. ( **A** ) Avaliação da possibilidade de sutura e posicionamento do guia tibial; ( **B** ) posicionamento de fio-guia; ( **C** ) criação de túnel ósseo com broca; ( **D** ) introdução e ajuste de agulha peridural no interior do túnel; ( **E** ) perfuração de menisco com agulha; ( **F** ) passagem de fio polipropileno (fio de transporte); ( **G** ) perfuração de menisco em nova posição; ( **H** ) visualização dos fios de transporte e posicionamento; ( **I** ) amarria dos fios de polipropileno no fio definitivo; e ( **J** ) tração do fio definitivo de poliéster pelo túnel ósseo.

**Fig. 2:** Aspecto final do reparo da raiz lateral em modelo sintético. Observe que o ponto de entrada é feito pela cortical anteromedial.

**Fig. 3:** Formato e posição dos túneis em relação ao ângulo de ataque no plano vertical em relação à superfície tibial. ( **A** ) Orifício com formato ovalado (menor inclinação); ( **B** ) orifício com formato circular (maior inclinação); e ( **C** ) orifício ovalado (+); orifício circular (*).

Terceiro passo: o ângulo de inclinação do guia terá influência na área de trabalho criada para se atingir o local desejado com a agulha epidural ( Fig. 3A,B ). Quanto menor o ângulo de inclinação (ângulo de ataque), maior a área criada. Com a diminuição do ângulo, o formato ovalado no orifício de saída permite pontos com alcance de até 1 cm ao utilizar brocas de 4,5 mm. O aumento do ângulo, por sua vez, faz com que o orifício se aproxime de uma circunferência. Seguindo o mesmo raciocínio, tem-se a projeção do dano à cartilagem e às estruturas adjacentes a ser causado neste processo. Em seguida, um fio-guia de 2,5 mm é posicionado, e sua posição é checada ( Fig. 1B ). Se for constatado bom posicionamento, faz-se a perfuração com broca canulada ( Fig. 1C ). A perfuração da porção articular tibial pode ser realizada em baixa rotação ou por rotação controlada de forma manual.

Quarto passo: a escolha do diâmetro da broca a ser usada na confecção do túnel transtibial também se orienta de acordo com o que foi exposto no terceiro passo. O aumento do diâmetro gerará maior área de trabalho, mas acarretará maior potencial de dano condral e de estruturas adjacentes. Sugere-se perfuração com broca canulada de 6 ou 7 mm na tentativa de reproduzir a área da anatomia das raízes meniscais posteriores medial e lateral, respectivamente. 7 O cirurgião deve estar atento não só ao ângulo de inclinação do guia tibial, como também ao diâmetro da broca a ser escolhida.

Quinto passo: introdução da agulha epidural (de calibre 18 × 150 mm) no túnel – ( Fig. 4A,B , 1D,G ). Esta agulha é maleável e mostrou-se adequada para percorrer túneis longos em posição oblíqua. A perfuração meniscal pode ser auxiliada pela utilização de um *probe* ou pinça *grasper* , o que permite o ajuste da posição do bordo meniscal a partir da tração gerada em direção ao túnel ou de sua angulação. Essa manobra evita a elevação do bordo durante a passagem da agulha. A manipulação do menisco com esses instrumentais permite modificar o ângulo de penetração da agulha, ajustando a amplitude do ponto de sutura. O controle da orientação do bisel da agulha pode ampliar o alcance do ponto ao girá-la em 180° em relação à primeira passagem da agulha, mantendo-a sempre apoiada nas paredes do túnel. Além disso, a perfuração deve ser realizada com o mandril no interior da agulha para evitar a entrada de tecido meniscal e, por consequência, uma perfuração inadequada.

**Fig. 4:** Demonstração da técnica para o menisco medial do joelho direito. ( **A,B** ) Inserção da agulha e passagem de dois fios de transporte (polipropileno); ( **C** ) fixação do fio definitivo (de poliéster) nas extremidades dos fios de transporte; ( **D** ) tração dos fios de transporte pelas porções distais no orifício tibial; e ( **E,F** ) aspecto final do reparo da raiz do corno posterior do menisco medial.

Sexto passo: após a perfuração, sugere-se a passagem de fio de polipropileno n° 2 no interior da agulha. ( Fig. 4A ). Em seguida, esse fio é capturado por um dos portais anteriores e trazido para fora do portal ( Fig. 4B ). Um reparo é preso à extremidade distal do fio. Repete-se esse passo para a passagem de outro ponto no mesmo bordo meniscal ( Fig. 4C ). As duas extremidades proximais dos fios de polipropileno, trazidas aos portais, são amarradas, cada uma, às extremidades de um fio de poliéster n° 2. ( Figs. 1C , 4C ). Uma tração leve é então realizada pelas extremidades distais dos fios de transporte na entrada do túnel tibial. Ao final, tem-se um ponto com configuração em “U” ( Figs. 4.D–F , 2B ) a ser fixado na cortical tibial. Sugere-se que pelo menos mais um ponto seja aplicado para aumentar a resistência e dividir a tensão local, o que diminui a chance de falha.

Sétimo passo: a fixação distal pode ser realizada conforme a disponibilidade de material. Como opções pode-se mencionar a utilização de arruela, poste, âncora com fios de alta resistência e ponto transósseo tibial. Nesse último passo, o cirurgião deve visualizar a posição de acomodação e o restabelecimento da anatomia meniscal antes de apertar o nó ( Fig. 1J ). Então, o nó é apertado com o joelho em flexão de 90°.

Ao final, realiza-se uma nova inspeção e remoção de detritos, caso existam. Testa-se então a estabilidade meniscal durante a flexo-extensão para o arco de movimento de 0 até 90° de flexão. Finalmente, o fechamento dos acessos é realizado de acordo com a preferência do cirurgião.

Na Fig. 5 , apresentamos as radiografias inicial e final de um dos casos de reparo da raiz posterior do menisco medial de um joelho direito. Na ocasião, os fios de sutura foram fixados distalmente com o auxílio de âncora óssea e fios de alta resistência.

**Fig. 5:** Radiografias de controle nos períodos pré e pós-operatório.

## Considerações Finais

A técnica de reparo da raiz meniscal posterior com agulha peridural por meio da combinação de portais anteriores e túnel transtibial mostrou-se factível e acessível. O seu uso pode eliminar a necessidade de portais posteriores e, com as estratégias descritas, permitir a restituição adequada da anatomia meniscal. Em nossa casuística, foram realizados doze reparos de raiz posterior meniscal por meio dessa técnica. Destes, nove foram realizados no menisco medial, e três, no menisco lateral. Em nenhum dos casos houve associação do reparo com algum procedimento concomitante (reconstrução ligamentar ou osteotomia). Além disso, nenhuma reabordagem foi necessária durante o seguimento de 12 meses, tendo os pacientes apresentado evolução satisfatória nesse período. Contudo, nenhum escore de avaliação foi aplicado durante o seguimento, sendo necessário um estudo adicional para avaliar a evolução no longo prazo.

É importante ressaltar que a execução da técnica demanda experiência do cirurgião na realização de manobras artroscópicas na região posterior do joelho, especialmente em casos de constrição do compartimento a ser manipulado. A dificuldade de manipulação de instrumentais nesse espaço pode elevar o tempo cirúrgico. Entre as maneiras possíveis de tornar a técnica mais fácil, é recomendável a utilização do garrote pneumático para reduzir o sangramento intra-articular e facilitar a visualização da articulação. Nas situações de constrição do compartimento, também pode-se realizar o *pie-crusting* do LCM para aumento do espaço de trabalho. Além disso, recomenda-se a utilização de um guia com alvo plano para facilitar o seu posicionamento e reduzir a chance de lesões iatrogênicas à cartilagem adjacente. Outras complicações possíveis são a laceração meniscal, que pode ocorrer após a tração do fio de sutura pelo orifício tibial, o que leva à falha do reparo, e a persistência de extrusão meniscal mesmo após o reparo, que pode ser corrigida com a feitura de um ponto de centralização para restabelecer a congruência do tecido meniscal.

Este estudo teve como objetivo trazer sugestões e detalhes técnicos para aumentar a taxa de sucesso do reparo da raiz meniscal posterior com a utilização de materiais de baixo custo. Assim, tem-se uma opção eficaz realizada com materiais acessíveis que pode ser utilizada em situações em que os recursos são limitados e em situações não previstas, para evitar a necessidade de um procedimento adicional por falta de instrumentais específicos no momento da cirurgia.
